# Transcriptional profiling of the effects of 25-hydroxycholesterol on human hepatocyte metabolism and the antiviral state it conveys against the hepatitis C virus

**DOI:** 10.1186/1472-6769-9-2

**Published:** 2009-01-16

**Authors:** John Paul Pezacki, Selena M Sagan, Angela M Tonary, Yanouchka Rouleau, Sylvie Bélanger, Lubica Supekova, Andrew I Su

**Affiliations:** 1 Steacie Institute for Molecular Sciences, The National Research Council of Canada, Ottawa, K1A 0R6 Canada; 2 Department of Biochemistry, Microbiology & Immunology, University of Ottawa, Ottawa, K1H 8M5, Canada; 3 Department of Chemistry, The Scripps Research Institute, La Jolla, CA 92037, USA; 4 Genomics Institute of the Novartis Research Foundation, San Diego, CA 92121, USA

## Abstract

**Background:**

Hepatitis C virus (HCV) infection is a global health problem. A number of studies have implicated a direct role of cellular lipid metabolism in the HCV life cycle and inhibitors of the mevalonate pathway have been demonstrated to result in an antiviral state within the host cell. Transcriptome profiling was conducted on Huh-7 human hepatoma cells bearing subgenomic HCV replicons with and without treatment with 25-hydroxycholesterol (25-HC), an inhibitor of the mevalonate pathway that alters lipid metabolism, to assess metabolic determinants of pro- and antiviral states within the host cell. These data were compared with gene expression profiles from HCV-infected chimpanzees.

**Results:**

Transcriptome profiling of Huh-7 cells treated with 25-HC gave 47 downregulated genes, 16 of which are clearly related to the mevalonate pathway. Fewer genes were observed to be upregulated (22) in the presence of 25-HC and 5 genes were uniquely upregulated in the HCV replicon bearing cells. Comparison of these gene expression profiles with data collected during the initial rise in viremia in 4 previously characterized HCV-infected chimpanzees yielded 54 overlapping genes, 4 of which showed interesting differential regulation at the mRNA level in both systems. These genes are PROX1, INSIG-1, NK4, and UBD. The expression of these genes was perturbed with siRNAs and with overexpression vectors in HCV replicon cells, and the effect on HCV replication and translation was assessed. Both PROX1 and NK4 regulated HCV replication in conjunction with an antiviral state induced by 25-hydroxycholesterol.

**Conclusion:**

Treatment of Huh-7 cells bearing HCV replicons with 25-HC leads to the downregulation of many key genes involved in the mevalonate pathway leading to an antiviral state within the host cell. Furthermore, dysregulation of a larger subset of genes not directly related to the mevalonate pathway occurs both in 25-HC-treated HCV replicon harbouring cells as well as during the initial rise in viremia in infected chimpanzees. Functional studies of 3 of these genes demonstrates that they do not directly act as antiviral gene products but that they indirectly contribute to the antiviral state in the host cell. These genes may also represent novel biomarkers for HCV infection, since they demonstrate an outcome-specific expression profile.

## Background

Hepatitis C virus (HCV) infection is a global health problem and a leading cause of liver disease in North America [1,2]. Previous studies have indicated that genes associated with cellular metabolic pathways are involved in the HCV life cycle. HCV infection causes the accumulation of lipid droplets (LDs) in patients' hepatocytes, a process called steatosis [3]. Both the HCV core protein and nonstructural protein NS5A are localized to LDs, replication complexes have been demonstrated to be located in LD-associated membranes, and that this recruitment is critical for producing infectious viruses [4-6]. Additionally, the expression of core protein in the liver of transgenic mice causes the development of steatosis [7].

Altering the host cell environment by using small molecules and other functional genomic approaches has been demonstrated to modulate HCV replication through diverse mechanisms [8-15]. Depletion of cholesterol using β-cyclodextrin disrupts the membranous web upon which HCV replication occurs [8]. Inhibitors of HMG-CoA reductase, the rate-controlling enzyme of the mevalonate pathway, like lovastatin and fluvastatin, also inhibit HCV replication [9,10]. In addition, small molecule inhibitors such as 25-hydroxycholesterol (25-HC) and cerulenin, both of which act at the mevalonate pathway, also inhibit HCV replication [11,12]. GGTI-286, an inhibitor of geranylgeranylation, inhibits HCV replication [13], and FBL2 has been implicated as a geranylgeranylated host protein required for HCV RNA replication [14]. The PPAR receptor antagonist 2-chloro-5-nitro-N-(pyridyl)benzamide also modulates HCV replication [15]. Collectively, these observations implicate a direct role of cellular lipid metabolism in the HCV life cycle and suggest that further insight into host cell metabolic determinants for the HCV life cycle may help to define new strategies for the development of antiviral therapeutics.

**Figure 1:**
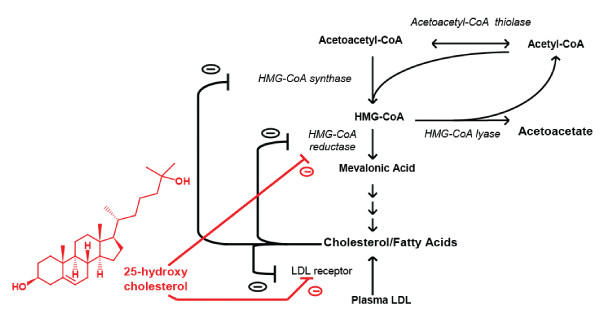
**The mevalonate pathway**. Schematic diagram of the mevalonate pathway with the targets of the inhibitor 25-hydroxycholesterol indicated.

Gene expression profiling has been used to study HCV-associated liver pathologies, including cirrhosis [16], fibrosis [17], and hepatocellular carcinoma (HCC) [18]. Other researchers have used microarrays to elucidate the mechanism(s) underlying IFN resistance [19] and the molecular basis for treatment failure of pegylated IFNα plus ribavirin [20-23]. The progression of HCV infection has also been studied by transcriptional profiling of liver biopsies in acutely infected chimpanzees that developed persistent infection, transient viral clearance, or sustained clearance, and common as well as outcome-specific changes in gene expression were observed [11]. Transient and sustained viral clearance was associated with the induction of IFNγ- induced genes, antigen processing and presentation genes, and genes associated with the adaptive immune response [11]. Host genes involved in lipid metabolism were also differentially regulated during the early stages of infection and these genes correlated with the outcome of infection [11]. These observations are particularly important given that HCV-derived steatosis is correlated with more rapid disease progression and reduced response to antiviral therapy clinically [24]. Herein we have performed gene expression profiling of Huh-7 cells harbouring HCV replicons in the absence and presence of the mevalonate pathway inhibitor 25-HC (Fig. 1) and examined which differentially regulated genes lead to an antiviral state in the replicon model. The changes in gene expression in the replicon model were compared with the gene expression profiles in HCV infected chimpanzees to evaluate their role in HCV pathogenesis.

## Results

**Figure 2:**
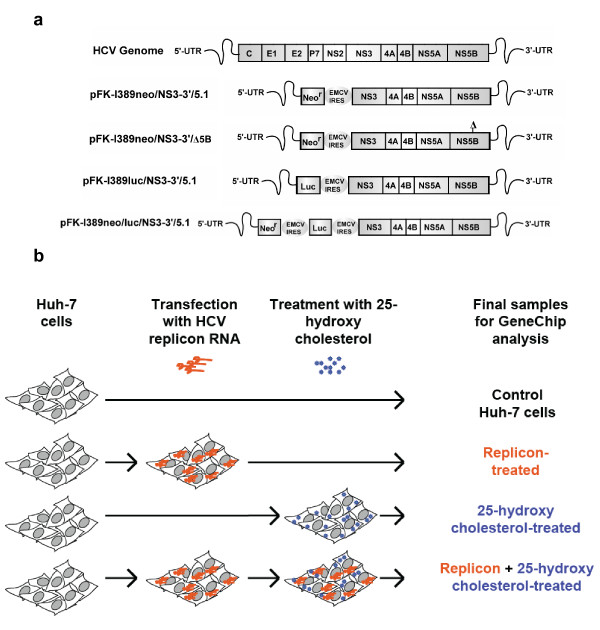
**HCV replicons and their use in transcriptional profiling with 25-hydroxycholesterol treatment**. **(a) **HCV genome and HCV subgenomic replicon constructs used in this study. **(b) **Experimental design for Affymetrix GeneChip experiments using HCV luc/NS3-3'/5.1 replicon RNA.

We performed transcriptome profiling using U133A Affymetrix high-density oligonucleotide arrays on RNA samples prepared from Huh-7 cells bearing HCV replicons in the presence of 25-HC, an inhibitor of the mevalonate pathway. The transcriptional profiling was carried out using cRNA prepared from four treatment groups: (1) naïve Huh-7 cells; (2) naïve Huh-7 cells treated with 25-HC; (3) Huh-7 cells transiently transfected with pFK-I_389_neo/NS3-3'/5.1 or replicon RNA from pFK-I_389_neo/NS3-3'/Δ5B replicon RNAs (Fig. 2a); and (4) Huh-7 cells transiently transfected with pFK-I_389_neo/NS3-3'/5.1 or pFK-I_389_neo/NS3-3'/Δ5B replicon RNAs and treated with 25-HC as indicated in Fig. 2b. The experiments were performed analogously to previous studies of antiviral small molecules on HCV replication [25-27]. As previously reported [8,11], the treatment of Huh-7 cells bearing HCV replicons with 25-HC caused a dose-dependent decrease in HCV replication at non-toxic concentrations as measured by firefly luciferase activity in Huh-7 cells transfected with HCV replicon RNA from pFK-I_389_luc/NS3-3'/5.1 (Fig. 2a).

Comparisons were conducted between each set of duplicate data to determine which genes were differentially expressed in 25-HC-treated Huh-7 cells and in the 25-HC-treated HCV replicon cells. The ratios of average expression for 25-HC treated Huh-7 cells/naïve Huh-7 cells were used to compute the fold change values for the 25-HC treated column Table 1. Similarly the 25-HC-treated HCV replicon column in Table 1 was computed from the ratio of average expression values from (replicon + 25-HC)/replicon samples. Transcriptome profiling of Huh-7 cells treated with 25-HC demonstrated that 69 genes were differentially regulated. Forty-seven genes were downregulated, 16 of which are clearly related to the mevalonate pathway (Table 1). A number of membrane proteins and oligonucleotide-binding proteins also appear to be downregulated (Table 1). By contrast, only 22 upregulated genes were observed upon treatment with 25-HC (Table 2). None of these genes are directly involved in the mevalonate pathway and there appears to be no functional relationship between the entries within Table 2. These observations suggest that 25-HC acts at the transcriptional level as a negative regulator of fatty acid and cholesterol biosynthesis [8,11,23], and that other changes in gene expression observed occur as secondary effects of 25-HC treatment. It is interesting to note that Huh-7 cells treated with both HCV replicon RNA and 25-HC did not show identical transcriptional profiles to cells treated analogously without HCV replicon RNA or to those only treated with HCV replicon RNA or replication-defective replicon RNA (pFK-I_389_neo/NS3-3'/Δ5B replicon RNA). Specifically, 5 genes were uniquely upregulated and 22 genes were uniquely downregulated in the 25-HC-treated HCV replicon harbouring cells (Table 3). Virtually all the genes in Table 3 are hypothetical transcripts or genes of unknown function. Collectively the global gene expression changes represented by Tables 1, 2, 3, represent the transcriptional program that leads to an antiviral state within the host cell hepatocyte, at least for the subgenomic HCV replicon. One or more of these genes may serve as prognostic markers for HCV treatment. Therefore, we performed further studies to investigate the importance of the observed gene expression changes in Tables 1, 2, 3 in other models for HCV pathogenesis.

**Table 1:** GeneChip analysis of downregulated genes after 25-HC treatment of Huh-7 cells and Huh-7 cells bearing HCV subgenomic replicons.^a^

	**Description^b^**	**Name**	**Accession #**	**Fold change 25-HC-treated**	**Fold change 25-HC-treated HCV replicon**
1	chromosome 14 clone BAC 507E23 map 14q24.3	B-ATF	AC007182	-4.1	-6.4
2	mRNA for cysteine protease		D55696.1	-3.9	---
3	RNA binding motif protein 5	RBM5	NM_005778.1	-3.1	---
4	tetratricopeptide repeat domain 3	TTC3	AI652848	-3.1	---
*5*	*insulin induced gene 1*	*INSIG1*	*BE300521*	*-2.8*	*-2.9*
			*BG292233*	*-2.2*	*-1.9*
			*NM_005542.1*	*-2.2*	*-2*
6	SON DNA binding protein	SON	AA664291	-2.4	-2.4
*7*	*3-hydroxy-3-methylglutaryl-Coenzyme A synthase 1 (soluble)*	*HMGCS1*	*NM_002130.1*	*-2.4*	*-2.4*
8	Similar to Nedd-4-like ubiquitin-protein ligase, clone MGC:2079		BC000108.1	-2.3	---
9	hypothetical protein DJ328E19.C1.1	DJ328E19.C1.1	BE732345	-2.3	---
10	Human DNA sequence from clone RP1-221C16 on chromosome 6		AL353759	-2.2	---
*11*	*low density lipoprotein receptor*	*LDLR*	*AI861942*	*-2.1*	*-3.3*
*12*	*3-hydroxy-3-methylglutaryl-Coenzyme A reductase*	*HMGCR*	*AL518627*	*-2.1*	*-1.9*
*13*	*fatty acid desaturase 1*	*FADS1*	*BG165833*	*-2.1*	*-1.7*
*14*	*sterol-C5-desaturase homolog*		*D85181.1*	*-2.1*	*---*
*15*	*Human lanosterol 14-alpha demethylase*	*CYP51P2*	*U40053*	*-2.1*	*-2*
16	FK506-binding protein 2	FKBP2	NM_004470.1	-2	-1.6
*17*	*apolipoprotein AI regulatory protein*	*ARP-1*	*M64497.1*	*-2*	*-3.5*
18	Homo sapiens HDCMC28P protein	HDCMC28P	NM_016649.1	-2	---
19	pleckstrin homology domain-containing, family A (phosphoinositide binding specific) member 1	PLEKHA1	NM_021622.1	-2	---
20	olfactory receptor, family 7, subfamily A, member 17	OR7A17	NM_030901.1	-1.9	2.4
*21*	*sterol-C4-methyl oxidase-like*	*SC4MOL*	*AV704962*	*-1.9*	*-2.5*
22	PRO0998	PRO0998	AF116616.1	-1.9	-2.6
23	ASMneg1-b1 immunoglobulin lambda chain VJ region	IGL	AF043584.1	-1.9	1.7
24	bone marrow protein BM037	BM037	NM_018454.1	-1.9	---
*25*	*3-hydroxy-3-methylglutaryl-Coenzyme A synthase 1*	*HMGCS1*	*BG035985*	*-1.9*	*-2.6*
*26*	*farnesyl diphosphate synthase*	*FDPS*	*NM_002004.1*	*-1.8*	*-1.8*
*27*	*mevalonate (diphospho) decarboxylase*	*MVD*	*AI189359*	*-1.8*	*-3.1*
28	kinesin family member 3C	KIF3C	NM_002254.1	-1.8	---
29	peptidyl-prolyl isomerase G (cyclophilin G)	PPIG	AW340788	-1.8	---
*30*	*acetyl-Coenzyme A acetyltransferase 2*		*BC000408.1*	*-1.8*	*-1.6*
*31*	*farnesyl-diphosphate farnesyltransferase 1*		*BC003573.1*	*-1.8*	*-2*
32	Homo sapiens Kruppel-like factor	LOC51713	NM_016270.1	-1.8	-2.4
33	prospero-related homeobox 1	PROX1	NM_002763.1	-1.7	---
34	cDNA FLJ13484 fis, clone PLACE1003888		AK023546.1	-1.7	---
35	M10098 Human 18S rRNA sequence			-1.7	---
36	chromodomain helicase DNA binding protein 4	CHD4	AI761771	-1.6	-2.1
37	topoisomerase (DNA) II alpha 170kDa	TOP2A	AU159942	-1.6	---
*38*	*7-dehydrocholesterol reductase*	*DHCR7*	*AW150953*	*-1.6*	*-2.2*
39	vesicle docking protein p115	VDP	BE875592	-1.6	-1.5
40	v-crk avian sarcoma virus CT10 oncogene homolog	CRK	NM_016823.1	-1.6	1.7
41	Nijmegen breakage syndrome 1 (nibrin)	NBS1	AI796269	-1.6	---
*42*	*farnesyl-diphosphate farnesyltransferase 1*	*FDFT1*	*AA872727*	*-1.6*	*-2.2*
43	cDNA FLJ11371 fis, clone HEMBA1000356		AK021433.1	-1.6	---
44	PAC clone RP5-979P20 from 7	NPM1P12	AC004941	-1.6	-1.5
45	erythrocyte membrane protein band 4.1-like 2	EPB41L2	BF511685	-1.5	1.9
46	activated leukocyte cell adhesion molecule	ALCAM	BF242905	-1.5	-1.7
47	protein kinase related to S. cerevisiae STE20	PAK4	NM_005884.2	-1.5	---
48	cDNA: FLJ21342 fis, clone COL02673.		AK024995.1	-1.5	---

^a ^Fold change was calculated from the average of two independent hybridizations on U133A Affymetrix GeneChip arrays compared with an average expression value from analogous hybridizations from control cells that were not treated with 25-HC. Fold change for 25-HC-treated column was determined from the ratio of expression in 25-HC treated Huh-7 cells/naïve Huh-7 cells and for the 25-HC-treated HCV replicon column from the ratio of expression values from (replicon + 25-HC)/replicon. ^b ^Italicized entries are known to be involved in the mevalonate pathway. Dashes (---) indicate genes whose fold-change in gene expression did not exceed 1.5-fold and hence were omitted from the analysis.

**Table 2:** GeneChip analysis of upregulated genes after 25-HC treatment of Huh-7 cells and Huh-7 cells bearing HCV subgenomic replicons.^a^

	**Description**	**Name**	**Accession #**	**Fold change 25-HC-treated**	**Fold change 25-HC-treated HCV replicon**
1	cytochrome P450-IIB	hIIB3	M29873.1	1.5	1.5
2	BTG family, member 2	BTG2	NM_006763.1	1.6	---
3	epithelial membrane protein 3	EMP3	NM_001425.1	1.6	---
4	gro-beta	GROB	M57731.1	1.6	---
5	S100 calcium-binding protein A6 (calcyclin)	S100A6	NM_014624.2	1.6	-1.6
6	KIAA0063 gene product	KIAA0063	NM_014876.1	1.7	---
7	acetyl-Coenzyme A carboxylase alpha	ACACA	BE855983	1.7	-2.9
8	HUMBT Chromosome 16p13.3 Exon Homo sapiens genomic clone h-80		L48784	1.7	-2.4
9	centrin, EF-hand protein, 2, clone MGC:12421		BC005334.1	1.8	2
10	intercellular adhesion molecule 1 (CD54), human rhinovirus receptor	ICAM1	AI608725	1.9	1.6
11	diubiquitin	UBD	NM_006398.1	1.9	1.5
12	cDNA DKFZp564P1263 (from clone DKFZp564P1263)		AL136653.1	1.9	3.4
13	H. sapiens hGDS mRNA for smg GDS.		X63465.1	1.9	2.1
14	actin related protein 23 complex, subunit 4	ARPC4	NM_005718.1	1.9	---
15	natural killer cell transcript 4	NK4	NM_004221.1	2	-1.6
16	ATX1 (antioxidant protein 1, yeast) homolog 1	ATOX1	NM_004045.1	2.2	---
17	Human DNA with a hepatitis B virus surface antigen (HBsAg) gene		M20919	2.7	1.6

^a ^Fold change was calculated from the average of two independent hybridizations on U133A Affymetrix GeneChip arrays compared with an average expression value from analogous hybridizations from control cells that were not treated with 25-HC. Fold change for 25-HC-treated column was determined from the ratio of expression 25-HC treated Huh-7 cells/naïve Huh-7 cells and for the 25-HC-treated HCV replicon column from the ratio of expression values from (replicon + 25-HC)/replicon. Dashes (---) indicate genes whose fold-change in gene expression did not exceed 1.5-fold and hence were omitted from the analysis.

**Table 3:** GeneChip analysis of gene expression changes after 25-HC treatment in HCV replicons.^a^

	**Description**	**Name**	**Accession #**	**Fold change 25-HC-treated**	**Fold change 25-HC-treated HCV replicon**
1	KIAA0552 gene product	KIAA0552	NM_014731.1	---	-3.5
2	solute carrier family 29 (nucleoside transporters), member 1	SLC29A1	NM_004955.1	---	-3
3	clone 25061		AF131777.1	---	-2.8
4	protein predicted by clone 23627 (HSU79266)	HSU79266	NM_013299.1	---	-2.5
5	hypothetical protein FLJ22390	FLJ22390	NM_022746.1	---	-2.3
6	Cip1-interacting zinc finger protein	CIZ1	NM_012127.1	---	-2.1
7	protein phosphatase 4, catalytic subunit, clone MGC:1882		BC001416.1	---	-2.1
8	CGI-28 protein	LOC51073	NM_015956.1	---	-2.1
9	mitochondrial Rho 2	MIRO-2	AI186666	---	-2.1
10	ribosomal protein L13	RPL13	AA789278	---	-2
11	chromosome 19, fosmid 39554	fos39554_1	AC004410	---	-2
12	hypothetical protein MGC3180	MGC3180	NM_024041.1	---	-1.9
13	EST, Weakly similar to FLJ14827 [H. sapiens]	FLJ14827	BE966197	---	-1.9
14	hypothetical protein DKFZp547L134	DKFZp547L134	NM_016553.1	---	-1.8
15	NADH dehydrogenase 1 alpha subcomplex, 7	NDUFA7	NM_005001.1	---	-1.6
16	hypothetical protein MGC8407	MGC8407	NM_024046.1	---	1.5
17	Homo sapiens full length insert cDNA YH77E09	YH77E09	AA928506	---	1.6
18	hypothetical protein FLJ12668	FLJ12668	NM_024997.1	---	2
19	cDNA FLJ10958 fis, clone PLACE1000444.		AU155621	---	2.9
20	hypothetical protein FLJ20556	FLJ20556	NM_017878.1	---	3.6
21	Similar to cadherin 6, type 2, K-cadherin (fetal kidney), clone MGC:1470		BC000019.1	---	4.1

^a ^Fold change were calculated from the average of two independent hybridizations on U133A Affymetrix GeneChip arrays compared with an average expression value from analogous hybridizations from control cells that were not treated with 25-HC. Fold change for 25-HC-treated column was determined from the ratio of expression 25-HC treated Huh-7 cells/naïve Huh-7 cells and for the 25-HC-treated HCV replicon column from the ratio of expression values from (replicon + 25-HC)/replicon. Dashes (---) indicate genes whose fold-change in gene expression did not exceed 1.5-fold and hence were omitted from the analysis.

We examined the transcriptional profiles of the genes from this study in three previously studied chimpanzees that were infected with HCV resulting in different outcomes of infection [28] and for whom genome-wide transcriptional analyses were performed during the course of infection [11]. Briefly, one chimpanzee successfully cleared the virus (sustained clearance, SC), one became persistently infected but initially transiently cleared the virus (transient clearance, TC), and the third chimpanzee developed an unrestrained persistent HCV infection (persistence, PS) in the absence of both an intrahepatic antiviral immune response and IFN-γ induction [28]. Genome-wide transcriptional profiling did suggest that the induction of both immunological and hepatocellular genes can influence the course and outcome of HCV infection [11]. Both the SC and TC chimpanzees displayed an altered lipid metabolism profile during the initial rise in viremia following inoculation with HCV as revealed by gene expression profiling, whereas the PS chimpanzee did not [11].

To determine which genes from our study were most relevant in the context of HCV infection in the chimpanzee model, we compared genes identified in Tables 1, 2, 3 with those of the chimpanzee data sets described previously [11]. To this end, we compared the replicon data sets (transient transfection) with the most representative chimpanzee data sets, those containing genes that changed significantly during the initial rise in viremia (described in the methods section), since both involve an initial host response to viral RNA and both involve the modulation of lipid metabolism genes. From the 69 differentially regulated genes identified here and the 257 genes derived from transcriptome profiling of infected chimpanzees during the initial rise in viremia [11], we identified 54 overlapping genes. These genes were further analyzed based on outcome-specific gene expression profiles, fold-induction in chimpanzees during the initial rise in viremia, function as determined by bioinformatically and using the SymAtlas at , and using the Ingenuity interaction software. The four genes selected for further study were prospero-related homeobox 1 (PROX1), insulin induced gene 1 (INSIG1), natural killer cell transcript 4 (NK4), and diubiquitin (UBD), Table 4.

**Table 4:** Comparative gene expression analysis of 25-HC-treated and HCV replicon-harboring Huh-7 cells with the changes in gene expression during HCV infection in chimpanzees with different outcomes of infection.^a^

		**Fold Change in Gene Expression over Naïve Huh-7 cells**				**Fold Change in Gene Expression during the Initial Rise in Viremia^c^**			
		
**Name**	**Accession #**	**Naïve Huh-7 + 25-HC**	**Huh-7 + luc/NS3-3'/Δ5B**	**Huh-7 + luc/NS3-3'/5.1**	**Huh-7 + luc/NS3-3'/5.1 + 25-HC**	**Naïve Chimpanzee**	**Sustained Clearance (SC)**	**Transient Clearance (TC)**	**Persistence (PS)**
PROX1	NM_002763.1	-1.7	-2.0	-2.0	-1.9	---	3.5	1.6	-2.3
INSIG1^b^	BG292233	-2.2	---	---	-1.8	---	1.6	2.9	---
	BE300521	-2.8	---	---	-2.8	N/A	N/A	N/A	N/A
	NM_005542.1	-2.2	---	---	-1.8	N/A	N/A	N/A	N/A
NK4	NM_004221.1	2.0	4.6	2.9	1.9	---	2.4	2.7	---
UBD	NM_006398.1	1.9	1.6	1.7	2.5	---	7.2	42.7	---

^a ^Comparisons were made with data collected from reference [11] for transcriptional profiles of 4 chimpanzees with different outcomes of viremia. See reference [11] for the details of the experiments for transcriptional profiling. The transcriptional profiling data from reference [11] was simply mined for the relevant data presented in this table; ^b ^Multiple INSIG1 Accessions correspond to the three INSIG1 probes; ^c ^Initial Rise in Viremia indicates gene expression levels at timepoint 3 after infection over timepoint 1 just prior to infection for each chimpanzee. Dashes (---) indicate genes whose fold-change in gene expression did not exceed 1.5-fold and hence were omitted from the analysis. N/A indicates transcripts that were not assessed in the chimpanzee gene expression profiles.

The PROX1 gene is a transcription factor expressed in the adult human liver that is involved in hepatocyte specification, proliferation, differentiation, and migration [29]. It is also involved in control of bile acid synthesis and cholesterol homeostasis *in vivo *[30]. Since HCV replication has been demonstrated to be linked with cellular lipid metabolism, and the PROX1 gene was found to have interesting expression patterns in previous gene expression studies carried out in chimpanzee [11,30], the PROX1 gene was chosen for further validation in the HCV replicon cells. In the replicon data set, PROX1 was downregulated in HCV replicon cells compared with naïve Huh-7 cells (Table 4). The PROX1 gene is downregulated in replicon-harbouring cells compared to naïve Huh-7 cells and represents a class of genes that may be negatively regulated during HCV replication. Interestingly, PROX1 is upregulated in TC and SC during the initial rise in viremia, but negatively regulated in PS (Table 4).

The INSIG-1 is highly expressed in the adult liver and plays a role in sterol homeostasis. INSIG-1 was downregulated in 25-HC-treated HCV replicon cells compared with HCV replicon-harbouring cells (Table 4). INSIG-1 represents a class of genes that are negatively regulated during HCV replication under metabolic conditions that are induced by the inhibitor of the mevalonate pathway, 25-HC. This transcript is also upregulated in an outcome-specific manner in the chimpanzee who experienced a clearance episode (SC and TC) but not in that which resulted in a persistent (PS) infection (Table 4).

The NK4 gene was chosen for further validation because its expression seems to be correlated with a clearance state in the chimpanzee and it was found to be upregulated in the 25-HC-treated HCV replicon cells compared with naïve Huh-7 cells treated with 25-HC (Table 4). Unlike PROX1 and INSIG1, NK4 represents a class of genes that are induced upon HCV replication and appears to be modulated by treatment with 25-HC.

Finally, the UBD gene was chosen for further validation because its expression was increased dramatically in the chimpanzees associated with sustained and transient clearance, 43-fold and 7-fold, respectively, and it was upregulated in 25-HC-treated HCV replicon cells compared with naïve Huh-7 cells treated with 25-HC (Table 4). UBD represents a class of genes that are induced during HCV replication when an antiviral state exists within the host cell, as demonstrated by the increase in expression in HCV replicon harbouring cells treated with 25-HC. This gene may serve as a prognostic marker for HCV infection.

**Figure 3:**
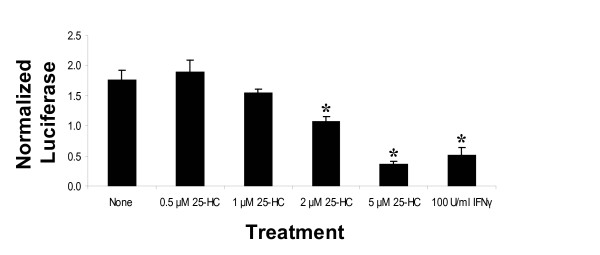
**Inhibition of HCV RNA replication by 25-hydroxycholesterol in a dose-dependent manner**. Activity data from Huh-7 cells transiently transfected with luc/NS3-3'/5.1 replicon RNA and treated with 25-HC or 100 U/ml IFNγ for 24 h. Bars represent the mean ± standard deviation of triplicate samples. An asterisk above a bar indicates a significant decrease in replication compared to untreated cells (*p *< 0.01).

**Figure 4:**
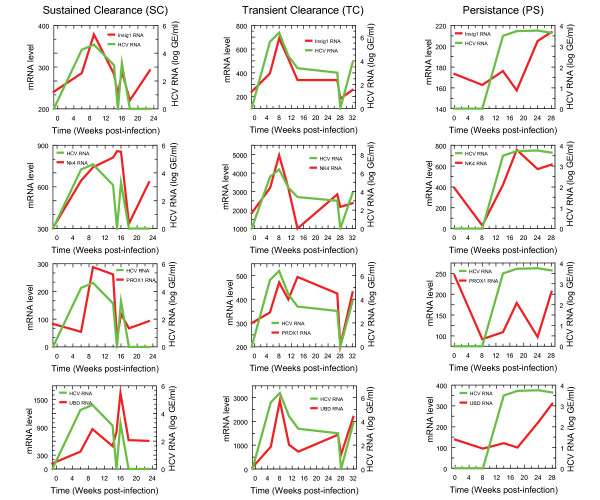
**Gene expression patterns for PROX1, INSIG-1, NK4, and UBD in HCV- infected chimpanzees**. Expression patterns of genes that are differentially regulated in HCV replicon cells during a time course of HCV infection for three infected chimpanzees displaying different outcomes of infection (SC, TC, and PS) [11]. Green lines represent log HCV RNA patterns, with values below the threshold for detection represented by zero.

Next we examined the overall gene expression patterns for these four genes (Table 4) in the TC, SC, and PS chimpanzees. The expression levels of the four genes in infected chimpanzees were plotted as a function of time alongside the HCV RNA levels measured in the chimpanzees [11] (Fig. 3). The comparative analysis between the chimpanzee and replicon data sets was done based on the first three chimpanzee time points, which corresponded to the initial rise in viremia [11]. However, the expression of the four genes of interest was determined and plotted for the entire measured time course of infection for the chimpanzees in Fig. 4. In general, the gene expression levels of the four genes paralleled the HCV levels in the chimpanzees that displayed sustained clearance (SC) and transient clearance (TC) of HCV. The overall gene expression levels were lowest in the persistently infected (PS) chimpanzee compared to the SC and TC chimpanzees. Also, the overall gene expression levels were higher for all four genes in the TC chimpanzee, which also had the highest viral titres (Fig. 4).

Expression of PROX1 in the HCV-infected chimpanzees early post-infection decreased slightly within the first six days for the SC chimpanzee, increased within the first eight days in the TC chimpanzee, and decreased within the first eight days in the PS chimpanzee (Fig. 4). Throughout the remaining course of infection, PROX1 levels roughly correlated with HCV levels in the SC and TC chimpanzees, while in the PS chimpanzee the PROX1 levels remained lower than the HCV levels. The expression level of INSIG-1 early post-infection increased in the SC and TC chimpanzees while it varied only slightly within the first eighteen days post-infection in the PS chimpanzee (Fig. 4). Thereafter, the INSIG-1 levels closely paralleled the HCV RNA levels in the SC and TC chimpanzees and rose to meet the HCV level in the PS chimpanzee. The NK4 levels during the initial rise in viremia in the chimpanzees paralleled the rising HCV levels in the SC and TC chimpanzees (Fig. 4). In contrast, in the PS chimpanzee the NK4 levels decreased within the first eight days of infection and then increased along with the rising HCV RNA level. Finally, the UBD pattern of expression basically paralleled the levels of HCV RNA in the SC and TC chimpanzees with some variation in the absolute levels of expression relative to that of the HCV RNA (Fig. 4). In the PS chimpanzee, the UBD expression level remained close to baseline levels in the first eighteen days post-infection, and then the UBD expression steadily increased during the remaining time course of HCV infection.

**Figure 5:**
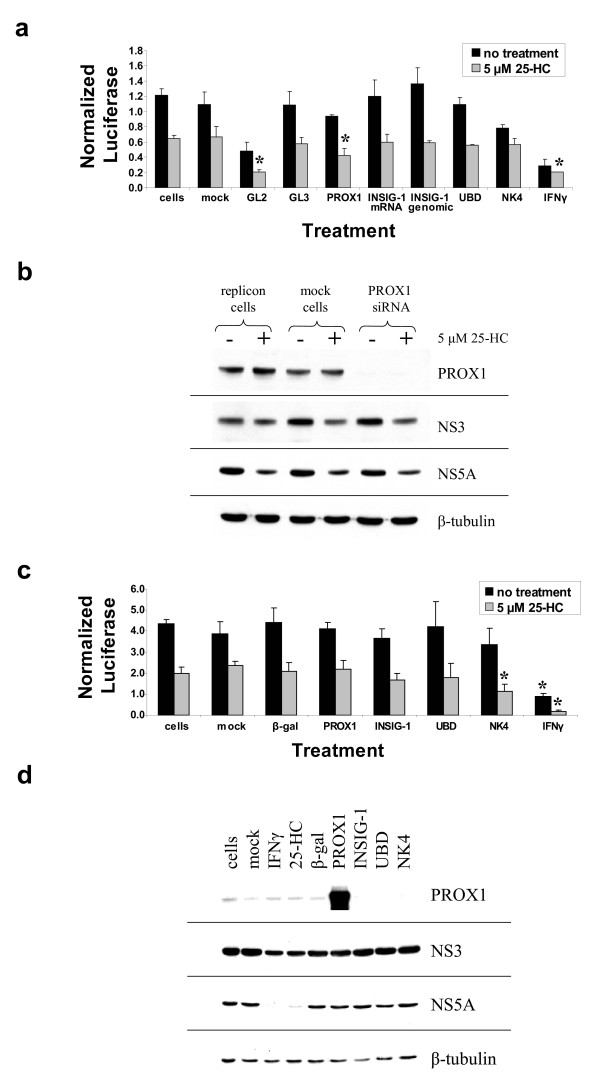
**The effect of 25-hydroxycholesterol and siRNA knockdown or gene overexpression on HCV RNA replication and translation**. Huh-7 cells stably expressing neo/luc/NS3-3'/5.1 or neo/NS3-3'/5.1 subgenomic replicons were transiently transfected with siRNA duplexes homologous to the genes of interest (A and B) or with expression plasmids for the genes of interest (C and D) in the absence or presence of 5 μM 25-hydroxycholesterol (25-HC) for 48 hours. After siRNA knockdown or overexpression of target genes, cell lysates were analyzed for luciferase activity (A and C) and western blot analysis of HCV non-structural proteins NS3 and NS5A as well as PROX1 (B and D). GL2 and GL3 siRNAs were used as controls for siRNA knockdown as described in the Methods. Mock refers to cells that were incubated with the transfection reagent but without siRNA or plasmid. As a positive control, cells were separately treated with 100 U/ml IFNγ. Values in (A) and (C) are the mean normalized relative light units (RLU) per mg/ml of protein ± standard deviation from triplicate samples. For the siRNA knockdown (A), an asterisk above a bar indicates a significant decrease in HCV replication compared to mock-transfected cells (GL2, PROX-1; *p *< 0.01) or untreated cells (IFNγ; *p *< 0.01) in the presence of 5 μM 25-HC. For the overexpression studies (C), an asterisk above a bar indicates a significant decrease in HCV replication compared to cells transfected with the backbone β-gal vector (NK4; p < 0.05) or the untreated cells (IFNγ; p < 0.01) when compared to the appropriate treatment condition (no treatment or 5 μM 25-HC treatment as indicated).

To ascertain the importance of the four genes for HCV replication, siRNA knockdown and overexpression studies were carried out in Huh-7 cells harbouring HCV replicon RNA. RT-PCR and northern blot analyses were used to confirm siRNA and overexpression vector function in each case (data not shown). Huh-7 cells stably expressing neo/luc/NS3-3'/5.1 HCV replicons [8,25-27] were treated with siRNAs against the four genes in the absence or presence of 25-HC (Fig. 5A). As controls, the HCV replicon cells were also treated with GL2 and GL3 siRNAs, a positive control that targets the luciferase gene expressed from the HCV replicon RNA and a negative control, respectively, in addition to IFNγ treatment [15]. Interestingly, although the PROX1 siRNA did not affect HCV replication on its own, it enhanced the inhibitory effect of treatment with 5 μM 25-HC when compared to mock-transfected replicon cells treated with 5 μM 25-HC (*p *< 0.01) as indicated (Fig. 5A). Overexpression of PROX1 in replicon cells did not affect HCV replication (Fig. 5C). Knockdown of INSIG-1 with homologous siRNAs did not affect HCV replication in replicon cells either in the absence or presence of 25-HC (Fig. 5A). Similarly, overexpression of INSIG-1 in replicon cells did not affect HCV replication (Fig. 5C). Although the transcriptional profiling showed that NK4 was upregulated in replicon cells treated with 25-HC, siRNA knockdown of NK4 did not affect HCV replication (Fig. 5A). However, overexpression of NK4 affected HCV replication in the presence of 25-HC treatment, causing a significant decrease in HCV replication compared to replicon cells that were transfected with the backbone β-gal control vector and treated with 25-HC (*p *< 0.05; Fig. 5C). From the profiling data, UBD was upregulated in 25-HC-treated replicon cells compared with naïve Huh-7 cells treated with 25-HC. However, neither knockdown of UBD using siRNA duplexes (Fig. 5A) nor overexpression of UBD (Fig. 5C) had an affect on HCV replication.

To determine if siRNA treatment or overexpression had any affect on the expression of the HCV nonstructural proteins NS3 and NS5A, Huh-7 cells stably expressing neo/NS3-3'/5.1 replicons were transiently transfected with siRNA or overexpression constructs and western blot analyses were conducted. As expected, siRNA knockdown of PROX1 gene expression resulted in complete loss of PROX1 protein expression (Fig. 5B). However, knockdown of PROX1 expression did not have any affect on NS3 or NS5A protein levels (Fig. 5B). Corresponding to their negative effect on HCV replication, IFNγ and 25-HC treatment of replicon cells caused a decrease in the levels of NS3 and NS5A, with the effect being more pronounced for NS5A as we had previously reported [25]. Interestingly, overexpression of INSIG-1 caused an increase in the levels of both HCV non-structural proteins (Fig. 5C). Overexpression of NK4 and UBD had no effect on the levels of HCV non-structural proteins (Fig. 5D).

## Discussion

Several examples now exist whereby HCV replication can be modulated or even cured by the targeting of host cell pathways rather than through the direct targeting of the virus [8,11,13-15,25-27]. Any perturbation of a host cell pathway that is harnessed or diverted by HCV during its life cycle has the potential to be an antiviral target, particularly if it is not an essential pathway to cell function. Small molecule probes have been used to block HCV replication by preventing the replication complexes from forming [8,11,13,14], by the selective inhibition of host cell kinases [26], by targeting nuclear hormone receptors [15], and by blocking protein folding pathways [27]. 25-HC has been established to yield an antiviral state within Huh-7 cells by modulating the mevalonate pathway [8,11,13,14]. To better understand the nature of the changes induced to the host cell, we performed global transcriptional profiling of Huh-7 cells and Huh-7 cells bearing HCV replicons. We identified unique sets of genes that are both up- and downregulated by 25-HC and established the transcriptional changes that lead to the inhibition of HCV replication in Huh-7 cells. By comparing these transcriptional profiles (Tables 1, 2, 3) with the gene expression profiles of HCV-infected chimpanzees we identified a number of transcripts that may be important for HCV pathogenesis. These genes may also be prognostic markers for disease progression and outcome that are indicative of the antiviral state of the host cell. From the 54 overlapping genes, we identified four genes for further study (Table 4), in an effort to establish a marker for HCV outcome that was linked to the transcriptional state of the host cell.

PROX1 is a highly conserved transcription factor that is expressed in hepatocytes and is involved in proliferation and migration [29]. It is also involved in the control of bile acid synthesis and cholesterol homeostasis *in vivo *[30]. The down-regulation of PROX1 in replicon cells compared to naïve Huh-7 cells suggests that PROX1 may negatively influence HCV replication but that this effect may only occur under certain metabolic conditions. The down-regulation of PROX1 was observed in the HCV replicon cells treated with 25-HC. Contrary to this, PROX1 expression was observed to rise early post-infection in the SC and TC chimpanzees (Fig. 4). The decreased expression of PROX1 in the chimpanzee with a persistent HCV infection suggests that PROX1 may be a predictor of the outcome of viremia. Dysregulation of PROX1 was also independently observed in another HCV-infected chimpanzee [31]. However, the fold changes observed for PROX1 may in fact be too small to act as a reliable prognostic marker for clinical applications.

INSIG-1 is highly expressed in the liver and plays an essential role in sterol homeostasis. INSIG-1 inhibits the sterol-dependent retention of the SCAP/SREBP complex in the ER and accelerates the sterol-stimulated degradation of HMG-CoA reductase, thus regulating the expression of cholesterol, fatty acids, and triglycerides [32,33]. Early post-infection, the expression level of INSIG-1 increased in the SC and TC chimpanzees and slightly decreased in the PS chimpanzee (Fig. 4), suggesting that this gene acts as an early metabolic predictor of outcome. Like 25-HC, INSIG-1 is an HMG-CoA reductase inhibitor and, therefore, it would be expected to decrease HCV replication. Since we did not observe any change in HCV replication with the INSIG-1 siRNA treatment, we can only conclude that the kinetics of siRNA knockdown of INSIG-1 were too slow to give rise to significant changes in HMG-CoA reductase during the course of the experiment, given that the INSIG-1 siRNA sequences had been previously validated [23]. Interestingly, overexpression of INSIG-1 caused an increase in the levels of HCV proteins NS3 and NS5A, which may be the result of a homeostatic response (Fig. 5D). The fold change values observed for INSIG1 may also limit its ability to serve as a prognostic marker.

NK4 was discovered by differential screening of a cDNA library derived from human NK cells that were activated by interleukin-2 [34]. Our observation that NK4 levels were increased early in viremia in the SC and TC chimpanzees (Fig. 4) is consistent with a previous report of increased NK4 expression in an HCV-infected chimpanzee [31]. NK4 was also upregulated in replicon cells treated with 25-HC (Table 2), suggesting that it may contribute to the suppression of replication caused by 25-HC. Taken together, the data suggests that NK4 may be a co-factor in inhibiting HCV replication, but it is not sufficient on its own to clear the virus in a persistent infection.

Diubiquitin (UBD, FAT10) is an alternative and ubiquitin-independent targeting mechanism for degradation by the proteasome [35]. UBD expression is inducible with IFNγ and TNF-α [36], both of which are well known antiviral cytokines. Additionally, previous gene expression profiling in HCV-infected chimpanzees identified differential regulation of proteasome genes [11,31,37]. From the profiling data derived from the HCV replicon cells, UBD was upregulated in 25-HC-treated replicon cells compared with naïve Huh-7 cells treated with 25-HC. This suggests that upregulation of proteasome degradation of HCV proteins may be an ancillary effect of 25-HC treatment. Neither knockdown of UBD using siRNA complexes nor overexpression of UBD had an affect on HCV replication in Huh-7 cells (Fig. 5), indicating that UBD is not a target for the development of potential therapeutic strategies for HCV infection. Rather, the very large fold change values observed, particularly in the large animal model for infection, suggests that it is a good candidate prognostic marker for an antiviral state within the host cell.

## Conclusion

Herein we have established the transcriptional changes that give rise to an antiviral state that inhibits HCV replication within Huh-7 cells after treatment with 25-HC. We identified four genes that can act as markers for HCV pathogenesis. The functional validation studies showed that none of these genes are likely to be useful as antiviral drug targets, they were identified as being linked to an antiviral state in the HCV replicon cells and in chimpanzee infections. Not surprisingly, INSIG1 and PROX1 transcriptional levels are related to regulation of the mevalonate pathway. The fact that these genes are differentially regulated in different models of HCV pathogenesis further supports their relevance. In the case of PROX1 and NK4, effects on HCV replication were observed in cell culture but only in certain metabolic states induced by the presence of 25-HC. The overall expression levels of all four genes were the lowest in the persistently infected chimpanzee which suggests that these genes may be predictors of the outcome of infection and might ultimately indicate a particular course of treatment on an individual patient basis. UBD appears to be the most promising since it was observed to have the largest changes in transcriptional levels in the chimpanzee model.

## Methods

### Cell culture

Cell monolayers of the human hepatoma cell line Huh-7 were grown in Dulbecco's modified minimal essential medium (DMEM) (Invitrogen, Burlington, Ontario) supplemented 10% with fetal bovine serum (FBS) (Cansera, Rexdale, Ontario) and with 100 nM non-essential amino acids (NEAA), 50 U/ml penicillin, and 50 μg/ml streptomycin. The medium was further supplemented with G418 to a concentration of 250 μg/ml for Huh-7 cells that stably expressed HCV replicons.

### HCV subgenomic replicons

The pFK-I_389_neo/NS3-3'/5.1, pFK-I_389_luc/NS3-3'/5.1, and pFK-I_389_neo/NS3-3'/Δ5B plasmids that contain HCV subgenomic replicons [8,22] were obtained from Ralf Bartenschlager (Institute of Hygiene, University of Heidelberg, Heidelberg, Germany). The replicons harbor either the selectable marker neo^R ^(pFK-I_389_neo/NS3-3'/5.1 and pFK-I_389_neo/NS3-3'/Δ5B) or the firefly luciferase gene (pFK-I_389_luc/NS3-3'/5.1) at the 5' end, but otherwise are identical and express HCV nonstructural proteins (NS3 to NS5B) from the encephalomyocarditis virus (EMCV) internal ribosomal entry site (IRES). The pFK-I_389_neo/NS3-3'/Δ5B plasmid carries an in-frame 10-amino acid deletion (Δ) encompassing the active site of the NS5B RNA polymerase that generates a replication defective replicon [22] (Fig. 2A). Additionally, a tricistronic HCV subgenomic replicon construct, pFK-I_389_neo/luc/NS3-3'/5.1, was also used in this study. This construct harbors both neo^R ^and firefly luciferase, with neo^R ^being translated from the HCV IRES, and the firefly luciferase and HCV nonstructural ORF translated from separate EMCV IRES motifs [8] (Fig. 2A). All plasmid constructs were confirmed by sequencing.

### *In vitro *transcription and transient transfection of replicon RNAs for total RNA extraction

*In vitro *transcripts of HCV replicon RNAs were generated using the MEGAscript™ kit (Ambion, Austin, Texas) according to the manufacturer's protocol and as described previously [8]. Huh-7 cells were seeded at 5 × 10^5 ^cells in 60 mm dishes to obtain cells at 70 to 80% confluency the next day. The second day, the cells were washed twice with 1× PBS, pH 7.4 (Gibco/Invitrogen, Burlington, Ontario), and once with serum- and antibiotic-free DMEM-NEAA. Transfection complexes were prepared by mixing 5 μg of *in vitro *transcribed replicon RNA (either luc/NS3-3'/5.1 or neo/NS3-3'/Δ5B) and 30 μl of DMRIE-C (Gibco/Invitrogen) in 1.5 ml of serum- and antibiotic-free DMEM-NEAA for each dish. The lipid-RNA complexes were immediately added to the washed cells and incubated for 4 h before commencing treatments. Mock-transfected cells received only the DMRIE-C reagent without any RNA.

### 25-HC and IFNγ treatments

Four hours post-transfection, the medium containing the lipid:RNA complexes was removed and replaced by DMEM-NEAA supplemented 10% with FBS ± 2 μM 25-HC. The cells were treated for 24 h before the lysates were prepared. As a positive control, some cells were treated with IFNγ (PBL Biomedical Laboratories, Piscataway, NJ) at a concentration of 100 U/ml. Mock-transfected cells were exposed to the treatment solvent, typically ethanol.

### Total RNA preparation for microarray experiments

Total RNA was prepared using the RNeasy^® ^Mini kit (QIAGEN, Mississauga, Ontario) and following the manufacturer's protocol. The RNA was quantified using the NanoDrop^® ^ND-1000 Spectrophotometer, and the quality of the RNA was verified using the RNA 6000 PicoLabChip kit (Agilent Technologies, Mississauga, Ontario).

### Gene expression analyses

Cellular cRNA samples were prepared and hybridized to high-density oligonucleotide arrays (Affymetrix U133A Human GeneChips) in the same manner as was done for the chimpanzeeanzee samples in our previous study [11]. Each sample was hybridized in duplicate. The neo/NS3-3'/Δ5B profiles were used as a negative control for HCV replication. Primary analyses were performed as described previously [11]. Briefly, the 116 genes identified as being differentially regulated in the replicon data set represent the logarithm-transformed genes that were deemed "present" and whose average difference exceeded 150 in at least one of the samples described above. Comparative analysis between the chimpanzee and replicon data sets was done based on the first three chimpanzee time points, which corresponded to the initial rise in viremia [11]. The post-infection time points corresponded to weeks -1, 6, and 9 for the SC chimpanzee, weeks -1, 5, and 9 for the TC chimpanzee, and weeks 0, 8, and 14 for the PS chimpanzee. The microarray data was submitted to GEO  with accession numbers GSM348423-GSM348433.

### Plasmids

Either the pCMV-SPORT6 vector (Invitrogen, Burlington, ON) or the pCMV-β vector (Clontech, Mountain View, CA) was used for gene overexpression. The pCMV-β vector had the β-galactosidase gene removed using *NotI *sites before subcloning the gene of interest. Dr. Anthony Orth [Genomics Institute of the Novartis Research Foundation (GNF), San Diego, CA] kindly provided the diubiquitin (UBD) construct in pCMV-SPORT6 by and the Natural Killer transcript 4 (NK4) construct in the pOTB7 Drosophila vector. The NK4 gene was subcloned into pCMV-β via a PCR-based strategy using *XhoI *and *NotI *restriction enzymes. A construct containing the prospero-related homeobox 1 (PROX1) gene was made by PCR-amplifying the gene from cDNA prepared from naïve Huh-7 cells, followed by a second round of PCR to add *NotI *sites to both ends, and subsequent subcloning into pCMV-β. The insulin-induced gene 1 (INSIG-1) construct in pCMV-SPORT6 was obtained from the ATCC (Manassas, VA).

### Treatment of HCV replicons with siRNAs or overexpression constructs

All siRNA duplexes were purchased from Dharmacon (Lafayette, CO). siRNAs homologous to GL2 luciferase and GL3 luciferase were used as positive and negative controls, respectively, as described previously [8]. Two custom siRNA duplexes homologous to the INSIG-1 mRNA sequence (UCA GUA UAC AUC CCC AGA U) and the INSIG-1 genomic sequence (UCA GUA UAC GUC CCC AGA U) were also purchased [23]. siRNA duplexes homologous to PROX1, NK4, and UBD were obtained from the siGENOME collection. For treatment of HCV replicons with siRNAs or overexpression constructs, Huh-7 cells stably expressing neo/luc/NS3-3'/5.1 HCV replicons were seeded at 5 × 10^4 ^cells per well in 24-well plates the day before transfection. The next day, 700 ng of plasmid DNA or 725 ng of siRNA was incubated for 20 min at room temperature with 1.4 μl (for DNA) or 1.45 μl (for siRNA) of Lipofectamine™ 2000 in 500 μl of serum- and antibiotic-free DMEM-NEAA. The DNA-lipid or siRNA:lipid complexes were then transferred onto the cells. Four hours later, the complexes were removed and 500 μl of DMEM-NEAA supplemented 10% with FBS was added to the cells and the different assays were done 48 h post-transfection. When a transient transfection was done to prepare whole cell lysates for western blot analyses, Huh-7 cells stably expressing neo/NS3-3'/5.1 replicons were seeded in 60 mm dishes with all quantities scaled up 10-fold.

### Luciferase reporter assay, total protein quantification, and western blot analysis

Replication of HCV replicon RNA was determined using a luciferase reporter assay as described previously [8]. Luciferase assay values were normalized by the total protein content of each sample and are shown as the mean ± standard deviation. The total protein content of whole cell lysates was quantified using the Bio-Rad DC Protein Assay (Bio-Rad, Mississauga, Ontario) as described previously [8]. Western blot analysis of whole cell protein lysates was carried out as reported previously [15] using the antibodies described below.

### Antibodies

The anti-PROX1 polyclonal antibody (Upstate, Charlottesville, VA) was used at a dilution of 1:4000 (v/v). Two monoclonal antibodies against the HCV nonstructural proteins NS3 and NS5A (Virostat, Portland, ME) were used at a 1:500 dilution (v/v). As a loading control, an anti-β-tubulin monoclonal antibody (BD Transduction Laboratories, Mississauga, Ontario) was used at a 1:1000 dilution (v/v). A goat anti-mouse HRP-conjugated secondary antibody (Jackson ImmunoResearch, West Grove, PA) was used at a dilution of 1:1000 (v/v) and a donkey anti-rabbit IgG (Jackson ImmunoResearch) was used at a dilution of 1:3000 (v/v).

### Statistics

Statistical analyses were carried out using GraphPad Prism software (version 4.01, GraphPad Software, San Diego, CA). Data was analyzed using one-way ANOVA. When overall differences were detected, Dunnett's post-test was performed to compare treatment groups to the appropriate control group. A *p *value of 0.05 or less was considered to be significant.

## Authors' contributions

JPP conceived the study and participated in its design and coordination and helped to draft and finalize the manuscript. SMS did the comparative analysis of the replicon and chimpanzee transcriptome data sets, cloned the NK4 gene, assisted in molecular genetic studies, and helped draft the manuscript. AMT performed data analysis, including the statistical analysis, and helped to draft the manuscript. YR carried out the molecular genetic studies and assisted with drafting the manuscript. SB cloned the PROX1 gene and assisted in molecular genetic studies. LS and AIS carried out the gene expression profiling of the replicon transcriptomes and AIS assisted SMS with the comparative analysis. All authors have read and approved the final manuscript.
